# Lower Respiration in the Littoral Zone of a Subtropical Shallow Lake

**DOI:** 10.3389/fmicb.2012.00434

**Published:** 2013-01-04

**Authors:** Ng Haig They, David da Motta Marques, Rafael Siqueira Souza

**Affiliations:** ^1^Programa de Pós-Graduação em Ecologia, Laboratório de Ecotecnologia e Limnologia Aplicada, Instituto de Pesquisas Hidráulicas, Universidade Federal do Rio Grande do SulPorto Alegre, Brazil; ^2^Laboratório de Ecotecnologia e Limnologia Aplicada, Instituto de Pesquisas Hidráulicas, Universidade Federal do Rio Grande do SulPorto Alegre, Brazil

**Keywords:** bacteria, macrophytes, phosphorus, chlorophyll *a*, humic substances, DOC, CO_2_

## Abstract

Macrophytes are important sources of dissolved organic carbon (DOC) to littoral zones of lakes, but this DOC is believed to be mostly refractory to bacteria, leading to the hypothesis that bacterial metabolism is different in littoral and pelagic zones of a large subtropical shallow lake. We tested this hypothesis by three approaches: (I) dissolved inorganic carbon (DIC) accumulation in littoral and pelagic water; (II) O_2_ consumption estimate for a cloud of points (*n* = 47) covering the entire lake; (III) measurement of O_2_ consumption and CO_2_ accumulation in dark bottles, *p*CO_2_ in the water, lake-atmosphere fluxes of CO_2_ (*f*CO_2_) and a large set of limnological variables at 19 sampling points (littoral and pelagic zones) during seven extensive campaigns. For the first two approaches, DIC and O_2_ consumption were consistently lower in the littoral zone, and O_2_ consumption increased marginally with the distance to the nearest shore. For the third approach, we found in the littoral zone consistently lower DOC, total phosphorus (TP), and chlorophyll *a*, and a higher proportion of low-molecular-weight substances. Regression trees confirmed that high respiration (O_2_ consumption and CO_2_ production) was associated to lower concentration of low-molecular-weight substances, while *p*CO_2_ was associated to DOC and TP, confirming that CO_2_ supersaturation occurs in an attempt to balance phosphorus deficiency of macrophyte substrates. Littoral zone *f*CO_2_ showed a tendency to be a CO_2_ sink, whereas the pelagic zone showed a tendency to act as CO_2_ source to the atmosphere. The high proportion of low-molecular-weight, unreactive substances, together with lower DOC and TP may impose lower rates of respiration in littoral zones. This effect of perennial stands of macrophytes may therefore have important, but not yet quantified implications for the global carbon metabolism of these lakes, but other issues still need to be carefully addressed before rejecting the general belief that macrophytes are always beneficial to bacteria.

## Introduction

Subtropical shallow lakes may present an important and differentiated set of conditions that make them ecologically distinct from other types of lakes. Because of the shallow mean depths and the benign climate conditions, macrophytes can colonize extensive areas and grow continuously throughout the year. These plants are important sources of organic matter to the littoral zones; in many cases they contribute more carbon than do the algae (Wetzel, 1992; Lauster et al., 2006) and in some systems, they sustain most of the bacterial production (Stanley et al., 2003).

However, macrophyte-derived carbon is believed to be mostly in the form of high-molecular-weight polymer-like compounds (Bracchini et al., 2006), poor in nitrogen and phosphorus contents (Hessen, 1992), and therefore refractory to bacterial consumption. Studies finding that the presence of macrophytes can be detrimental to bacterioplankton are starting to accumulate. Wu et al. (2007) found lower bacterial diversity in submersed macrophyte-dominated areas than in areas with no macrophytes in the large Lake Taihu, China. Rooney and Kalff (2003) surveyed nine lakes with different percentages of macrophyte coverage, and found a significant decrease in the bacterial respiration rate with increasing macrophyte coverage. In southern Brazil, bacterial metabolism and biovolume were found to be lower in the littoral (covered with macrophytes) than in the pelagic zones of subtropical shallow lakes (They et al., 2010). If bacterial metabolism can be lower in the presence of macrophytes, there is a contradiction with the common belief that macrophyte-derived carbon always benefit bacteria, and it is reasonable to hypothesize that carbon cycling can be affected in the littoral zones of lakes that are extensively colonized by these plants.

Estimates of bacterial respiration are essential to assess the balance of a lake’s metabolism because they provide a direct assessment of the fate of carbon within the system, reflecting its transfer from the organic to the inorganic pool (Jahnke and Craven, 1995). It is well established that plankton respiration increases with chlorophyll, phosphorus, and organic carbon concentrations in lakes (Pace and Prairie, 2005). The effect of primary production (in terms of chlorophyll *a*) on bacterial production has long been recognized, even in systems dominated by terrestrial-carbon inputs (Kritzberg et al., 2005). Phosphorus can also play a role. High rates of respiration and CO_2_ supersaturation have been associated with high humic content of the carbon pool, because they are an attempt to balance phosphorus deficiency in these low-nutrient-content substrates (Hessen, 1992). Moreover, the ratio of phosphorus to labile DOC can control partitioning between bacterial respiration and production (Cimbleris and Kalff, 1998; Smith and Prairie, 2004; del Giorgio and Newell, 2012). DOC concentration, in turn, is also positively associated with CO_2_ supersaturation in lakes (Prairie et al., 2002).

In the present study, we tested the hypothesis that there is a difference in the general metabolism of carbon in the littoral versus pelagic zones in the large, subtropical shallow Lake Mangueira, through: (a) an experiment to measure dissolved inorganic carbon (DIC) accumulation in littoral and pelagic water; (b) measurement of respiration in a cloud of points covering littoral and pelagic zones; and (c) extensive temporal measurement of respiration, *p*CO_2_, CO_2_ fluxes, and limnological variables related to bacterial-carbon metabolism in a smaller cloud of points, also covering the whole lake.

## Materials and Methods

### Study site

**Figure 1:**
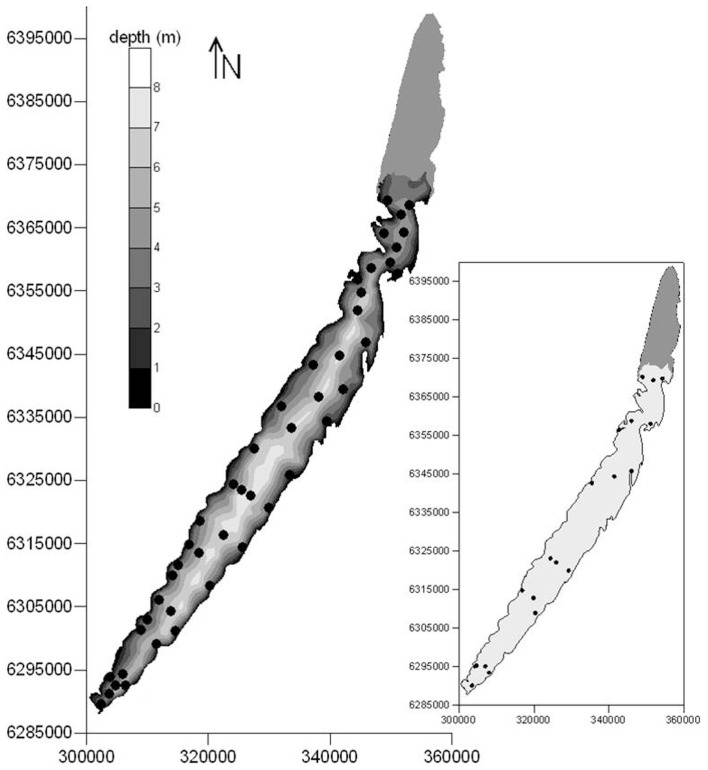
**Locations of sampling points for respiration rates sampled in March 2009 (main map), and extensive sampling campaigns of respiration rates and limnological variables carried out in 2010–2011 (inset) in Lake Mangueira, a large shallow lake in subtropical southern Brazil**. Maps are georeferenced in UTM coordinate system.

Lake Mangueira (80,800 ha, southern Brazil, state of Rio Grande do Sul) is a large, shallow, freshwater subtropical coastal lake. It is surrounded by extensive belts of wetlands, located primarily in the northern and southern areas. The DIC accumulation experiment (to test question a above) was done in the southernmost part of the lake, and the two clouds of points (to test questions b and c above) covered the entire lake (Figure 1).

### DIC accumulation in littoral × pelagic water

Samples of pelagic and littoral water were collected and incubated for 14 days for determination of the cumulative DIC, in April–May 2007. We retrieved vials at days 0, 1, 3, 5, 7, 10, and 14, in triplicates (*n* = 42). The incubation vials (sterile 40 mL clear borosilicate vials, open top with silicone/PTFE septum and teflon-lined screw caps) were carefully filled with no headspace (Farjalla et al., 2001) and incubated at room temperature (∼20°C) in the dark. DIC was analyzed in a Total Organic Carbon (TOC) Analyzer (Shimadzu VCPH). Bulk respiration rates were considered an approximation of bacterial respiration, since several pilot experiments with different water fractions showed no significant differences between the bulk and bacterial fractions after filtration in MN 640d Macherey–Nagel paper filters (2.0–4.0 μm mean retention size). This filtration has been extensively tested and successfully excludes ciliates, metazoans, and most flagellates.

### Respiration in littoral × pelagic zones

A large set of measurements of respiration rates, covering the entire Lake Mangueira, was taken in March 2009. The water was collected in the littoral and pelagic zones (littoral *n* = 21; pelagic *n* = 26) and the water was incubated in dark bottles, for 6 days in the laboratory (Wetzel and Likens, 2000). Dissolved oxygen was measured by a TOGA/SRI gas chromatograph (purge trap injector for water samples and TCD/HID detectors). Oxygen measurements were converted to carbon, assuming a molar conversion factor of 1.0 (del Giorgio et al., 1997). Results were expressed in μg C L^−1^ h^−1^, assuming a constant respiratory rate.

Nineteen points in the littoral and pelagic zones (littoral *n* = 12; pelagic *n* = 7) covering the entire lake were collected for several limnological variables during seven campaigns: pH, Abs250, Abs365, Abs250:365, total phosphorus (TP), total nitrogen (TN), chlorophyll *a*, DOC, DIC, O_2_ consumption, CO_2_ accumulation, *p*CO_2_ in the water, and CO_2_ fluxes (*f*CO_2_). With the exceptions of *p*CO_2_ and CO_2_ fluxes, all variables were collected in May, August, and November 2010 and March, May, August, and November 2011. *p*CO_2_ and *f*CO_2_ were estimated in February, May, August, and November 2010 and March, June, and August 2011.

Surface water was taken using a horizontal sampling bottle. pH was measured with a potentiometer (Wetzel and Likens, 2000). Abs250 and Abs365 refer to absorbance at 250 and 365 nm respectively, in 1 cm-optical path length quartz cuvettes. The former is proportional to the low-molecular-weight compounds, while the latter is proportional to the high-molecular-weight compounds present; and Abs250:365 is the ratio Abs250:Abs365 (Strome and Miller, 1978; Lindell et al., 1995). Samples for TN and TP were frozen in 1 L polyethylene bottles, and these nutrients were measured by colorimetry (Mackereth et al., 1989). Chlorophyll *a* was quantified through cold ethanol extraction (Jespersen and Christoffersen, 1987). DOC and DIC samples were collected in 30 mL precombusted (450°C for 1 h) amber glass bottles. DOC samples received a few drops of $H_{3}PO_{4}^{-3}$, and DIC and DOC were analyzed in the same TOC analyzer (Shimadzu VCPH).

Oxygen consumption and CO_2_ accumulation were determined by incubation in glass serum bottles. The bottles were kept inside a thermal box (approximately 23°C) and taken to the laboratory, totaling 120 h of incubation. O_2_ was measured in a TOGA/SRI gas chromatograph, and CO_2_ indirectly from measurements of DIC and pH after correction of temperature and ionic strength (Stumm and Morgan, 1996). The measurements were made at the beginning and at the end of incubation. The ionic strength was estimated from the electrical conductivity of the water measured in the field, using the following conversion formula (Snoeyink and Jenkins, 1980):

$$IS\approx 1.6x10^{-5}\times \;Conductivity$$

where *IS* is the ionic strength and the water conductivity is measured in μS cm^−1^.

The *p*CO_2_ in the water was calculated based on the Law of Henry, with the following equation:

$$pCO_{{}_{2}}^{w}=\frac{\alpha_{0}DIC}{K_{H}}$$

where α_0_ corresponds to the fraction of DIC as CO_2_ and *K_H_* is the dissolution constant of CO_2_ expressed in moles L^−1^ atm^−1^ corrected for temperature in kelvin (*T_k_*) after the equation of Weiss (1974):

$$\text{ln }K_{H}\left(CO_{2}\right)=-58.0931+90.5069\;\left(\frac{100}{T_{k}}\right)+22.294\text{ ln }\left(\frac{T_{k}}{100}\right)$$

The flux of CO_2_ (ƒCO_2_, mmoles C m^−2^ d^−1^) between atmosphere and lake was estimated with the following equation (MacIntyre et al., 1995):

$$fCO_{2}=k_{x}\left(pCO_{2}^{atm}-pCO_{2}^{w}\right)$$

where $pCO_{2}^{atm}$ is the partial pressure of CO_2_ in the atmosphere (for 380 ppm), $pCO_{2}^{w}$ is the partial pressure of CO_2_ estimated for the surface layer of the lake, and *k_x_* is the coefficient of mass transfer (cm h^−1^), given by the following empirical relationship:

$$k_{x}=k_{600}{\left(\frac{S_{c}}{600}\right)}^{-x}$$

where *x* is equal to 0.66 for winds <3.0 m s^−1^ and equal to 0.5 for winds >3.0 m s^−1^, *S_c_* is the number of Schmidt for CO_2_, which is dependent on temperature (°C), according to the following relationship (Wanninkhof, 1992):

$$S_{c}\left(CO_{2}\right)=1911.1-118.11T+3.4527T^{2}-0.04132T^{3}$$

where *k*_600_ is estimated from wind velocity (Cole and Caraco, 1998):

$$k_{600}=2.07+\left(0.215\;U_{10}^{1.7}\right)$$

where *U*_10_ is the wind velocity (m s^−1^).

Data from the Santa Vitória do Palmar meteorological station (www.inmet.gov.br), located 7.0 km from the lake, were used to obtain air temperature and wind velocity.

### Statistical treatment

Differences between DIC accumulation in dark (littoral) and clear (pelagic) water samples and among different time periods for each type of water were tested with one-way non-parametric ANOVA (NPMANOVA in the univariate mode) test (Anderson, 2001). The dissimilarity measure used was Euclidean distance, with 9999 permutations. Software: PAST 2.14 (Hammer et al., 2001).

For the respiration data set, the mean rates of respiration in the littoral (*n* = 21) and pelagic zones (*n* = 26) were tested for difference with ANOVA after natural-log transformation (correction for normality). The association between rates of respiration (also transformed) of the cloud of points and the distances from the nearest margin was tested by linear regression (Software R 2.15.0, R Development Core Team, 2012).

The data from the O_2_ consumption, CO_2_ accumulation, *p*CO_2_, CO_2_ fluxes, and limnological variables obtained in the seven sampling campaigns were *z*-score standardized within each month and split into two categories, based on the distance from the nearest shore: littoral (<1000 m) and pelagic (≥1000 m). This threshold was based on the mean distance of the samples from the shore. Differences between the littoral and pelagic zones were tested for each variable individually (pH, Abs250, Abs365, Abs250:365, TP, TN, chl *a*, DOC, O_2_, CO_2_, *f*CO_2_) by one-way NPMANOVA (Anderson, 2001). We used the software PAST v. 2.14 (Hammer et al., 2001) with Euclidean distance and 9999 permutations. *A posteriori* contrast tests used Bonferroni correction (*n* × *p*, where *n* is the number of comparisons and *p* is the *p* value obtained by permutation) implemented in the software PAST 2.14 (Hammer et al., 2001).

The explanatory power of abiotic variables (pH, Abs250, Abs365, Abs250:365, TP, TN, chl *a*, and DOC) on O_2_ consumption, CO_2_ accumulation, *p*CO_2_, and *f*CO_2_ was assessed by binary recursive partitioning (regression tree) with the help of the R 2.15.0 (R Development Core Team, 2012) package {rpart} (Therneau et al., 2012) and following Crawley (2007). For O_2_ consumption and CO_2_ accumulation all months were included (*n* = 133); for CO_2_ and *f*CO_2_, given the unbalance in sampling months, February (2010) and June (2011) were excluded (*n* = 95).

## Results

### DIC accumulation experiment

The NPMANOVA test indicated that DIC was higher in the pelagic zone (23.24 ± 1.54 mg C L^−1^) than in the littoral zone (16.82 ± 1.72 mg C L^−1^), *pseudo*-*F* = 163.0, *p* = 0.0001. There was no change in DIC with time: Littoral zone: *pseudo*-*F* = 0.957, *p* = 0.524; pelagic zone: *pseudo*-*F* = 0.685, *p* = 0.777.

### Respiration clouds of points

**Figure 2:**
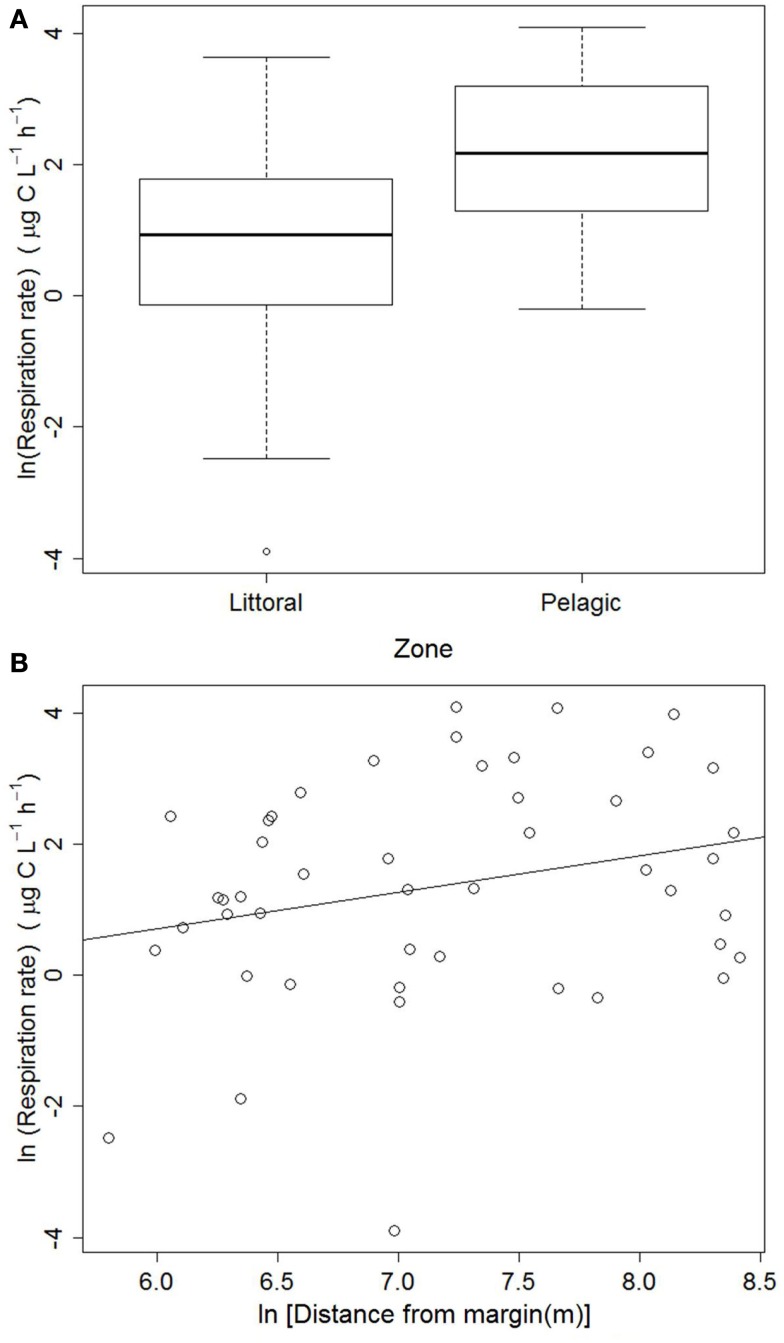
**Respiration rates in littoral (*n* = 21) and pelagic (*n* = 26) zones (A) and regression of respiration rates against the distance to the nearest shore (B) in Lake Mangueira, sampled in March 2009**.

The respiration data collected from the entire lake revealed that respiration rates were significantly lower in the littoral zone: *F*(1, 45) = 10.778, *p* < 0.002 (Figure 2A). The regression between respiration rates and distance from the nearest shore was marginally significant: ln(Respiration) = −2.648 + 0.559[ln(Distance)], Adjusted *R*^2^ = 0.046, *p* = 0.079 (Figure 2B).

The NPMANOVA with all months revealed a significantly higher Abs250:365 in the littoral zone and a significantly higher TP and chl *a* in the pelagic zone. DOC was marginally significantly higher in the pelagic zone (Tables 1 and 2).

**Table 1:** **Mean ± standard deviation of individual environmental variables for all sampling campaigns and by month for the cloud of points assessed in Lake Mangueira from 2010 to 2011**.

Variable		2010–2011	2010				2011			
		All months	February	May	August	November	March	May	August	November
pH	*L*	7.83 (±0.28)^a^	–	**7.94 (±0.27)^a^**	7.44 (±0.06)^a^	8.17 (±0.08)^a^	7.86 (±0.31)^a^	7.95 (±0.18)^a^	7.79 (±0.16)^a^	7.64 (±0.15)^a^
	*P*	7.84 (±0.27)^a^	–	**7.75 (±0.15)^b^**	7.44 (±0.04)^a^	8.16 (±0.15)^a^	7.98 (±0.26)^a^	7.85 (±0.14)^a^	7.89 (±0.10)^a^	7.83 (±0.29)^a^
Abs250	*L*	0.093 (±0.033)^a^	–	0.069 (±0.007)^a^	0.100 (±0.022)^a^	0.091 (±0.012)^a^	0.087 (±0.031)^a^	**0.102 (±0.055)^a^**	**0.112 (±0.054)^a^**	0.088 (±0.007)^a^
	*P*	0.086 (±0.021)^a^	–	0.074 (±0.018)^a^	0.106 (±0.022)^a^	0.103 (±0.021)^a^	0.084 (±0.016)^a^	**0.070 (±0.015)^b^**	**0.078 (±0.018)^b^**	0.087 (±0.010)^a^
Abs365	*L*	0.016 (±0.008)^a^	–	0.008 (±0.002)^a^	0.023 (±0.005)^a^	0.017 (±0.005)^a^	0.014 (±0.006)^a^	**0.017 (±0.014)^a^**	0.014 (±0.008)^a^	0.021 (±0.003)^a^
	*P*	0.017 (±0.008)^a^	–	0.010 (±0.003)^a^	0.026 (±0.010)^a^	0.022 (±0.009)^a^	0.015 (±0.005)^a^	**0.009 (±0.003)^b^**	0.014 (±0.004)^a^	0.020 (±0.002)^a^
Abs250:365	*L*	**6.49 (±2.57)^a^**	–	**9.11 (±1.18)^a^**	4.42 (±0.46)^a^	5.49 (±0.88)^a^	6.54 (±1.31)^a^	7.09 (±1.62)^a^	**8.48 (±4.59)^a^**	4.31 (±0.54)^a^
	*P*	**5.83 (±1.69)^b^**	–	**7.71 (±1.07)^b^**	4.24 (±0.77)^a^	4.96 (±0.89)^a^	5.72 (±0.89)^a^	8.08 (±1.45)^a^	**5.69 (±0.77)^b^**	4.44 (±0.72)^a^
TP (mg L^−1^)	*L*	**0.033 (±0.020)^a^**	–	**0.017 (±0.005)^a^**	0.020 (±0.006)^a^	0.025 (±0.007)^a^	**0.026 (±0.005)^a^**	0.027 (±0.006)^a^	0.042 (±0.015)^a^	**0.076 (±0.008)^a^**
	*P*	**0.037 (±0.018)^b^**	–	**0.025 (±0.007)^b^**	0.030 (±0.019)^a^	0.030 (±0.009)^a^	**0.031 (±0.008)^b^**	0.030 (±0.010)^a^	0.042 (±0.016)^a^	**0.069 (±0.004)^b^**
TN (mg L^−1^)	*L*	0.33 (±0.14)^a^	–	0.43 (±0.08)^a^	0.24 (±0.03)^a^	0.25 (±0.12)^a^	0.25 (±0.09)^a^	0.20 (±0.10)^a^	0.51 (±0.04)^a^	0.43 (±0.10)^a^
	*P*	0.35 (±0.16)^a^	–	0.43 (±0.07)^a^	0.26 (±0.04)^a^	0.20 (±0.14)^a^	0.29 (±0.07)^a^	0.23 (±0.09)^a^	0.55 (±0.15)^a^	0.48 (±0.07)^a^
chl *a* (μg L^−1^)	*L*	**3.03 (±1.09)^a^**	–	**2.76 (±1.18)^a^**	**3.43 (±1.01)^a^**	2.48 (±0.69)^a^	**2.82 (±0.88)^a^**	2.87 (±1.31)^a^	**3.04 (±0.71)^a^**	3.82 (±1.49)^a^
	*P*	**3.89 (±1.49)^b^**	–	**4.21 (±1.63)^b^**	**5.11 (±1.86)^b^**	3.14 (±1.23)^a^	**3.99 (±1.63)^b^**	3.41 (±1.25)^a^	**3.98 (±1.11)^b^**	3.38 (±0.93)^a^
DOC (mg L^−1^)	*L*	**1.89 (±1.23)^a^**	–	2.13 (±1.01)^a^	2.56 (±1.22)^a^	1.90 (±0.83)^a^	2.28 (±2.44)^a^	1.54 (±0.48)^a^	1.79 (±0.87)^a^	1.05 (±0.44)^a^
	*P*	**2.38 (±1.51)^b^**	–	2.86 (±0.94)^a^	2.57 (±0.79)^a^	1.92 (±0.40)^a^	4.02 (±2.24)^a^	2.45 (±1.64)^a^	1.80 (±1.23)^a^	1.05 (±0.83)^a^
DIC (mg L^−1^)	*L*	15.03 (±4.36)^a^	–	13.59 (±1.08)^a^	13.49 (±1.86)^a^	11.44 (±1.06)^a^	12.70 (±2.20)^a^	12.18 (±1.85)^a^	19.97 (±4.39)^a^	21.83 (±0.46)^a^
	*P*	14.41 (±3.99)^a^	–	13.73 (±1.09)^a^	12.30 (±1.41)^a^	11.02 (±0.78)^a^	12.25 (±2.38)^a^	12.72 (±0.95)^a^	16.48 (±3.13)^a^	22.35 (±0.56)^a^
O_2_ (cons.; mg L^−1^)	*L*	2.57 (±1.64)^a^	–	2.98 (±1.57)^a^	3.11 (±1.46)^a^	1.95 (±2.22)^a^	1.53 (±0.56)^a^	4.24 (±2.22)^a^	1.94 (±0.48)^a^	2.21 (±0.60)^a^
	*P*	2.74 (±1.52)^a^	–	3.59 (±2.22)^a^	3.53 (±1.52)^a^	2.43 (±1.42)^a^	1.86 (±0.55)^a^	3.26 (±1.65)^a^	2.27 (±1.12)^a^	2.23 (±0.89)^a^
CO_2_ (accum.; mg L^−1^)	*L*	5.81 (±4.14)^a^	–	6.36 (±6.32)^a^	4.21 (±4.90)^a^	**3.97 (±2.62)^a^**	4.78 (±2.49)^a^	6.26 (±2.86)^a^	7.50 (±2.80)^a^	7.61 (±5.34)^a^
	*P*	5.60 (±4.40)^a^	–	2.84 (±5.41)^a^	6.10 (±5.90)^a^	**6.92 (±3.90)^b^**	4.43 (±2.12)^a^	5.29 (±5.13)^a^	8.03 (±2.48)^a^	5.62 (±3.34)^a^
								**June**		
*p*CO_2_ (μatm)	*L*	345 (±161)^a^	192 (±71)^a^	484 (±66)^a^	467 (±85)^a^	479 (±65)^a^	317 (±100)^a^	100 (±17)^a^	372 (±127)^a^	–
	*P*	368 (±181)^a^	192 (±38)^a^	453 (±53)^a^	531 (±99)^a^	566 (±134)^a^	337 (±64)^a^	98 (±10)^a^	401 (±120)^a^	–
*f*CO_2_	*L*	−10.1 (±41.6)^a^	−71.0 (±24.6)^a^	34.4 (±22.5)^a^	10.1 (±10.4)^a^	26.9 (±18.5)^a^	−14.1 (±21.0)^a^	−54.5 (±3.5)^a^	−2.7 (±26.9)^a^	–
(mmol C m^2^ d^(1^)	*P*	−5.6 (±45.2)^a^	−70.7 (±13.7)^a^	23.8 (±18.3)^a^	18.0 (±12.3)^a^	51.1 (±37.3)^a^	−9.7 (±13.3)^a^	−55.1 (±2.2)^a^	3.2 (±26.0)^a^	–

*Different superscripts and bold indicate significant (*p* < 0.05) or marginal (0.05 < *p* < 0.1) differences between littoral and pelagic zones for each month (not among months), except for the global test with 2010–2011, where the difference of littoral and pelagic zones of all months was tested. Details of NPMANOVA in Table 2. Groups coded according to the distance from the nearest shore: Littoral (*L*, < 1000 m) × Pelagic (*P*, ≥ 1000 m)*.

**Table 2:** **Non-parametric MANOVA (NPMANOVA) analysis on individual environmental variables for all campaigns and by month for the cloud of points sampled in lake Mangueria from 2010 to 2011**.

Year	Month	Variable	NPMANOVA	
			Output	Effect
2010–2011	All	Abs250:365	F* = 5.20, *p* = 0.023	*L* > *P*
		TP	F* = 4.02, *p* = 0.044	*P* > *L*
		Chl *a*	F* = 12.44, *p* < 0.001	*P* > *L*
		DOC	F* = 3.11, *p* = 0.083*	*P* > *L*
2010	May	pH	F* = 3.95, *p* = 0.066*	*L* > *P*
		Abs250:365	F* = 7.02, *p* = 0.0172	*L* > *P*
		TP	F* = 9.24, *p* = 0.010	*P* > *L*
		Chl *a*	F* = 4.15, *p* = 0.062*	*P* > *L*
	August	Chl *a*	F* = 4.78, *p* = 0.042	*P* > *L*
	November	CO_2_	F* = 3.14, *p* = 0.093*	*P* > *L*
2011	March	TP	F* = 2.91, *p* = 0.098*	*P* > *L*
		Chl *a*	F* = 3.06, *p* = 0.090*	*P* > *L*
	May	Abs250	F* = 3.67, *p* = 0.042	*L* > *P*
		Abs365	F* = 3.29, *p* = 0.044	*L* > *P*
	August	Abs250	F* = 4.03, *p* = 0.045	*L* > *P*
		Abs250:365	F* = 4.41, *p* = 0.013	*L* > *P*
		Chl *a*	F* = 3.97, *p* = 0.0597*	*P* > *L*
	November	TP	F* = 7.14, *p* = 0.015	*L* > *P*

Groups coded according to the distance from the nearest shore: littoral (*L*, < 1000 m) × Pelagic (*P*, × ≥ 1000 m). In bold: *p* < 0.05; *, 0.05 < *p* < 0.1). F*, *pseudo*-*F*.

The NPMANOVA by each month separately revealed that TP and chl *a* were frequently higher in the pelagic zones (May, August 2010 and March, August, and November 2011). Less frequent, but also consistent was higher Abs250 and Abs250:365 in littoral zones (May 2010 and May and August 2011). Punctually, littoral zones also showed higher pH (May 2010) and DIC (August 2011), while the pelagic zone showed higher CO_2_ concentration (November 2010; Tables 1 and 2).

**Figure 3:**
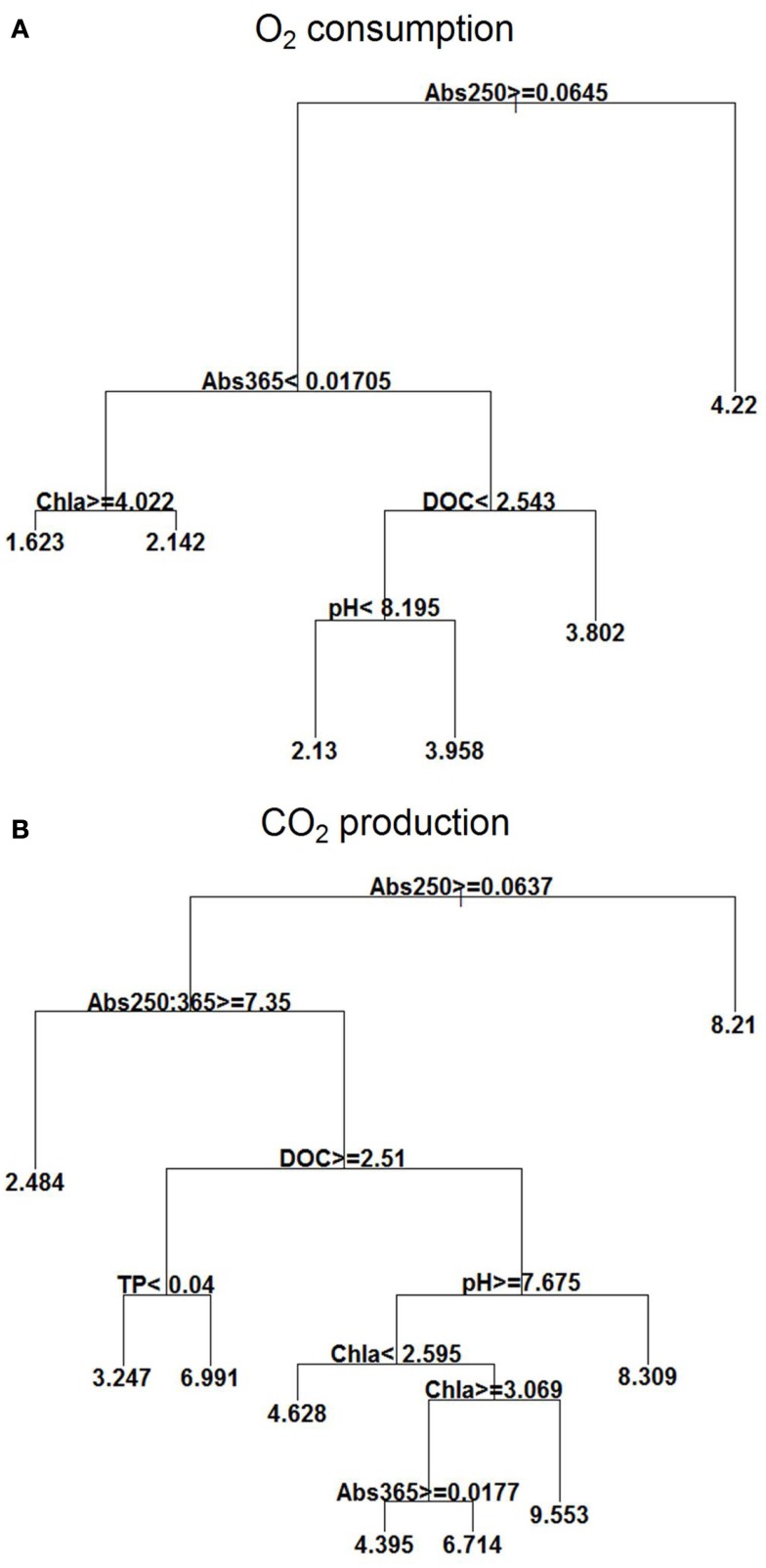
**Explanatory power of environmental variables assessed by regression tree on O_2_ consumption (mg L^−1^) (A) and CO_2_ (mg L^−1^) production (B) with the extensive campaigns data (*n* = 133)**. Means in terminal leafs. Notice that the partitioning criteria above nodes applies to the left branches (< or ≥), while the complement (≤ or >) applies to the right branches.

The regression trees revealed that the Abs250 was the major environmental variable influencing O_2_ consumption, CO_2_ production, and *p*CO_2_, while DOC influenced most the *f*CO_2_ (Figures 3A,B).

For O_2_ consumption, the highest mean occurred at Abs250 < 0.064; at Abs250 ≥ 0.064, there was also influence of Abs365, chl *a*, DOC, and pH, with high O_2_ consumption occurring at Abs365 ≥ 0.017 and DOC ≥ 2.54 mg L^−1^. At lower concentrations of DOC (<2.54 mg L^−1^), O_2_ consumption was also high when the pH was ≥8.2. Lowest O_2_ consumption occurred at Abs365 < 0.017 and chl *a* concentrations ≥4.02 μg L^−1^ (Figure 3A).

CO_2_ production was also high at Abs250 < 0.064, but reached higher means at Abs250 ≥ 0.064), Abs250:365 < 7.35, DOC < 2.51 mg L^−1^, pH ≥ 7.67, and high chl *a* concentrations (between 2.59 and 3.07 μg L^−1^). At DOC concentrations <2.51 mg L^−1^ and low pH < 7.67, the mean was also high. The low values, however, occurred at Abs250 ≥ 0.064, and Abs250:365 ≥ 7.35 (Figure 3B).

**Figure 4:**
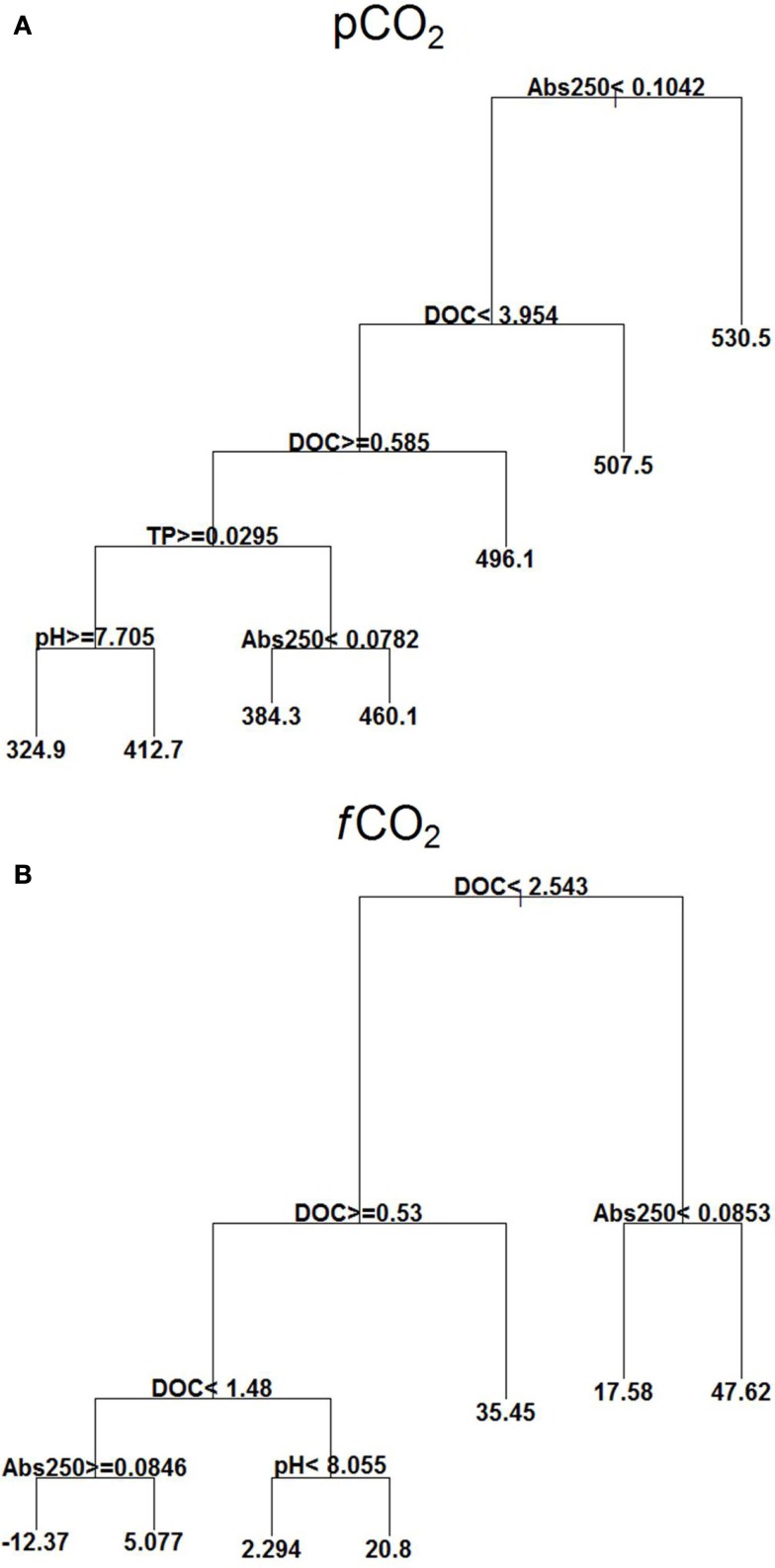
**Explanatory power of environmental variables assessed by regression tree on *p*CO_2_ (μatm) (A) and *f*CO_2_ (mmol C m^2^ d^−1^) (B) with extensive campaigns data (*n* = 95)**. Means in terminal leafs. Notice that the partitioning criteria above nodes applies to the left branches (< or ≥), while the complement (≤ or >) applies to the right branches.

*p*CO_2_ showed high values at Abs250 ≥ 0.104. At lower Abs250 (<0.104), there was also influence of DOC, TP, pH, and Abs250, with lowest values occurring at DOC between 0.58 and 3.95 mg L^−1^, TP ≥ 0.029 mg L^−1^, and pH ≥ 7.70 (Figure 4A).

*f*CO_2_ showed a strong relationship with DOC concentrations. At DOC ≥2.54 mg L^−1^, and Abs250 ≥ 0.0853 it was found the highest positive value of *f*CO_2_. A negative mean was found with values of DOC between 1.48 and 2.54 mg L^−1^ and at Abs250 ≥ 0.0846 (Figure 4B).

## Discussion

Our results showed that respiration can be lower in the littoral zone, based on the consistently lower DIC in water collected from this zone in incubation assays. In the first whole-lake survey, we found significantly lower respiration rates in these zones and a marginal (*p* = 0.079) increase of respiration rate with distance to the nearest shore. In the extensive sampling, this was not consistently found throughout all months. Marginally (*p* = 0.093) lower CO_2_ accumulation was detectable in at least 1 month (November 2010) in the littoral zones. However, significantly higher DIC occurred in the littoral zones in August 2011. These differences are possibly associated with differences in environmental conditions between these zones, as shown by consistently higher chlorophyll *a* and TP, marginally higher DOC (*p* = 0.083) and significantly lower Abs250:365 in the pelagic zone, as shown by NPMANOVA analyses on the entire data set (all months). The regression tree analyses confirmed that higher O_2_ consumption and CO_2_ production are associated to low Abs250, high DOC, and high Abs365. *p*CO_2_ and *f*CO_2_, at their turn, were higher at high Abs250, DOC, and TP.

Our results showed contrasting patterns. The DIC accumulation experiment and the extensive sampling of 2009 supported our hypothesis that respiration is lower in the littoral zone than in the pelagic zone. However, apart from the marginally higher CO_2_ accumulation in November 2010 in the pelagic zone, the extensive sampling of 2010–2011 did not show such striking evidence in support of the hypothesis. This could be due to the smaller number of points sampled, but also the result of intensive mixing of Lake Mangueira by wind (Cardoso et al., 2012), which makes it difficult to detect abrupt differences in respiration in the field, as indicated by several marginal relationships found. This could have been crucial because of the smaller set of sampling points.

One hypothesis for the lack of detection of DIC accumulation is that in areas with an extensive coverage of macrophytes, like southern Mangueira, the macrophyte influence might reach pelagic zones as well. We unfortunately do not have enough data gathered yet to envisage how, why, and when this occurs. We nevertheless did observe that DIC was on average higher in the pelagic zone, likely due to very low accumulation rates (not statistically significant in <14 days). When we extend our perspective (spatially and temporally) to the whole lake and for different years and seasons, we see a different picture, where we do detect respiration (even though still low) enough to put in evidence differences between littoral and pelagic zones. Another important point to take into consideration is that lake Mangueira is of marine origin (former closed estuary) with significant accumulation of sea shell banks. This explains the high background levels of DIC in the lake, and could have masked measurements of bacterial respiration based on DIC. This also suggests that it is not the best method for this purpose, and may have been the cause for the disagreement between the patterns of DIC and O_2_/CO_2_ respiration rates observed.

As stated, in Lake Mangueira, rates of respiration are extremely low, and it is striking that it is impossible to detect respiration in dark bottles over a period shorter than 5 days. The mean respiration rates in the littoral (1.34 mmol O_2_ m^−3^ h^−1^) and pelagic zones (1.43 mmol O_2_ m^−3^ h^−1^) are closer to the lower range presented in the extensive compilation of plankton respiration rates (0.029 to 6.73 mmol O_2_ m^−3^ h^−1^) by Pace and Prairie (2005). This is also confirmed in a carbon-basis comparison, as the rates of respiration in this study (Littoral zone: Mean: 1.469 μg C L^−1^ h^−1^, range: 0.008–6.241 μg C L^−1^ h^−1^; Pelagic zone: Mean: 6.540 μg C L^−1^ h^−1^, range: 0.250–23.718 μg C L^−1^ h^−1^) are also close to the lower range reported in other systems: Delaware Bay and salt marshes (range: 2.53–13.14 μg C L^−1^ h^−1^; del Giorgio and Newell, 2012) and 20 Quebec lakes (range: 0.168–2.138 μg C L^−1^ h^−1^).

The differences in respiration in the littoral and pelagic zones of Lake Mangueira may reflect differences in the responses of bacterial metabolism to the following drivers: (I) quantity and bioavailability of DOC; and (II) phosphorus limitation. Our results supported both hypotheses, as indicated by the lower chl *a*, DOC, and TP, and the higher proportion of low-molecular-weight compounds (in terms of Abs250 and Abs250:365) in the littoral zone found in many cases.

The generally low DOC bioavailability in the form of humic substances (Münster and Chróst, 1990) indicates that a large proportion of dissolved carbon in Lake Mangueira is refractory to bacterial consumption. When compared to the values reported by Amado et al. (2006) for an Amazonian river and a stream, the content of humic substances in Lake Mangueira is 6 times higher. The humic content is at least 4 times higher than for values reported for the mesohumic Lake Sjättesjön (Lindell et al., 1995), or even as much as 12–17 times higher than reported for the Gutierrez River (all comparisons on the basis of the Abs250:365 nm ratio; Pérez et al., 2003).

DOC is generally high in densely vegetated areas (Wetzel, 1992; Reitner et al., 1999); however, we found higher DOC in the pelagic zone, along with chl *a*, suggesting the importance of algal carbon to the system. This is in accordance with the long-recognized bacterial dependence on algal production (Cole, 1982), particularly because the more labile algal DOC sustains higher bacterial growth efficiency (Kritzberg et al., 2005). This was evident by the greater phytoplankton biomass concentrated (higher chlorophyll *a*) in the pelagic zone at the end of winter (August 2010), which was respired by bacteria with the rising temperatures in spring (highest CO_2_ accumulation found in November 2010; Table 1).

The higher concentration of low-molecular-weight substrates in the littoral zone can also imply less availability of substrates (Amon and Benner, 1996). Contrariwise, these substrates are supposedly more easily taken up by bacteria, but they may be more refractory to consumption. Tranvik (1990) found higher bacterial production per unit of carbon in high-molecular-weight DOC than in low-molecular-weight DOC in lakes with different humic content in Sweden. In another study, Amon and Benner (1996) found higher bacterial production and respiration in high-molecular-weight (HMW, >1 KDa) than low-molecular-weight (LMW, <1 KDa) DOC. Based on these results, the authors proposed the size-reactivity continuum model, which predicts that the major path of degradation goes from large, highly reactive to small, highly recalcitrant molecules. If this hypothesis is broadly applicable to many ecosystems, it suggests that in Lake Mangueira the compounds lixiviated by the macrophytes may undergo some degradation, but accumulate as dissolved, unreactive LMW compounds with very low degradation rates in the littoral zones, in agreement with the higher Abs250:365 and lack of change in the DIC with time in the incubation experiment. Our results strongly support this hypothesis, as higher O_2_ consumption, and CO_2_ production were found for low Abs250 and high Abs365 or conversely lower O_2_ consumption, and CO_2_ production were found when the ratio Abs250:365 was too high.

The lower TP found in the littoral zone can be attributed to the generally low watershed load and also the great competitive advantage of macrophytes in taking up this nutrient. As noted by Vidal et al. (2011), not only carbon, but the carbon: phosphorus ratio controls bacterial production and respiration in lakes, and even a situation of labile DOC accumulation can occur under phosphorus limitation (Vadstein et al., 2003). In fact, increased bacterial density in the presence of macrophytes after phosphorus amendment has been described (Huss and Wehr, 2004; Joniak et al., 2007; Morozova et al., 2011). An interesting result was the lower TP in the pelagic zone in November 2011, which could be due to depletion of this nutrient in the pelagic zone during summer (Table 1); however, no similar depletion occurred in 2010, and cannot be assumed as a pattern based on our data. The regression trees indicated an effect of TP when DOC is high; low TP is associated to low CO_2_ production, while the opposite situation also applies, supporting the hypothesis that low respiration can be associated to low TP. This is expected to generate lower *p*CO_2_ in littoral zones where TP is lower; however a difference in *p*CO_2_ between littoral and pelagic zones was not detected. The regression tree revealed that *p*CO_2_ was low when TP was high in samples of high DOC. This is in agreement with low phosphorus leading to CO_2_ supersaturation in order to balance this nutrient deficiency (Hessen, 1992), and suggests that this also occurs in lake Mangueira. As CO_2_ concentration is only part of *p*CO_2_, other factors affecting *p*CO_2_ equilibrium need to be taken into account, and this may be the cause for the apparent contradiction between CO_2_ production and *p*CO_2_ predicted from TP.

Levels of *p*CO_2_ were in general lower than in other lakes with similar or even lower TP content: mean around 660 μatm (range: 130–1010 μatm) for a range of Quebec lakes with variable trophic content; mean around 1 036 μatm (range: 1–20 249 μatm) for a comprehensive compilation of data for 1835 lakes distributed worldwide (Cole et al., 1994). These levels are far above those found in this study. This could be explained by the generally low DOC concentration in Lake Mangueira (mean around 2.2 mg L^−1^), because it has been demonstrated that lakes are net heterotrophic (supersaturated with CO_2_) above concentrations of 4–6 mg L^−1^ DOC (Prairie et al., 2002). This contrasts with the generally accepted view that most aquatic systems are net heterotrophic and function as net sources of CO_2_ to the atmosphere (Cole et al., 1994; Duarte and Prairie, 2005), which makes Lake Mangueira (particularly the littoral zone) an exception to this general rule.

**Figure 5:**
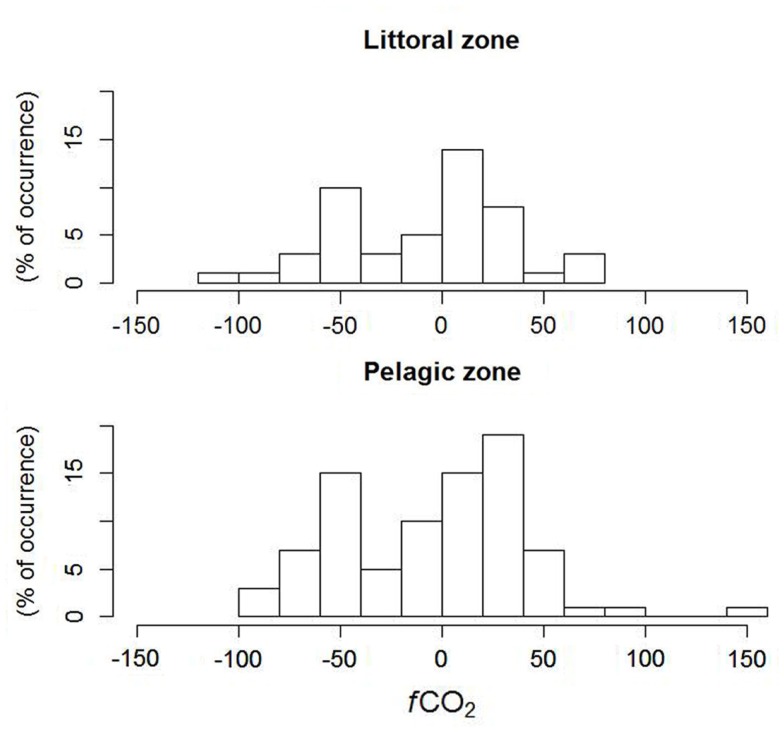
**Histogram of CO_2_ fluxes between atmosphere and lake surface in littoral (*n* = 12) and pelagic (*n* = 7) zones in Lake Mangueira, sampled in 2010–2011**.

The analysis of the frequency of occurrence (histogram) of classes of *f*CO_2_ between the lake surface and atmosphere in the littoral zone showed a higher frequency of positive (net influx of CO_2_ to the lake), while the pelagic zone showed a higher frequency of negative values (net efflux of CO_2_ from the lake) of *f*CO_2_, even though there was no difference between the means in the littoral and pelagic zones. A closer inspection of the fluxes showed that they are very close to zero, and the lake functions as a sink of CO_2_, especially during winter and spring (May to November), when the *f*CO_2_ becomes close to zero or even positive (Figure 5). The regression tree showed that high, positive *f*CO_2_ was associated to higher DOC and Abs250, contrary to the expectation that higher DOC is associated to higher CO_2_ supersaturation and hence net efflux of CO_2_ from the lake; negative *f*CO_2_ was associated to intermediate values of DOC and high Abs250. However, more direct evidence needs to be gathered in order to draw more solid associations.

Besides main effects of DOC and nutrients, other possible explanations for low respiration in the littoral zone in Lake Mangueira have also been hypothesized: formation of hydrogen peroxide by UV radiation, release of allelochemicals by macrophytes, and limitation by micronutrients, e.g., iron and silicate. UV exposure of macrophytes products of degradation in Lake Mangueira has been demonstrated to decrease bacterial production, possibly by formation of H_2_O_2_ (They et al., submitted). The release of allelopathic substances by (mainly submersed) macrophytes is widely known (Gross et al., 2007; Mulderij et al., 2007), and has been considered as a possible explanation for lower bacterial diversity (Wu et al., 2007) and metabolism (They et al., 2010) in areas extensively colonized by macrophytes. We have data on Fe and silicate from the extensive campaigns that were not included in the results because they are out of objectives of this study. Based on these data, there is no evidence of iron limitation, since the values found in the littoral zones (0.325 ± 0.253 mg L^−1^) and pelagic zones (0.349 ± 0.248 mg L^−1^) are far above concentrations reported to be limiting in other systems, like, e.g., open ocean (<5.6 × 10^−6^ mg L^−1^; Oliver et al., 2004) or lake Erie (1.7 × 10^−4^–1.09 × 10^−2^ mg L^−1^; North et al., 2007). Silicate concentrations are far above those reported to be limiting (2 μm) for diatoms (Egge and Aksnes, 1992) in littoral (100.92 ± 23.99 μm) and pelagic zones (100.04 ± 23.83 μm), and thus there no evidence of possible impacts on bacterial supply of organic substrates derived from silicate limitation of phytoplankton.

One important and final consideration is that we found support for our hypotheses by different, independent methods: one-time DIC accumulation, extensive one-time sampling and extensive many-times sampling by O_2_ consumption, and CO_2_ production measurements. Each method has its own limitations, as we believed was crucial in the DIC experiment; had we employed a longer incubation period, we could have seen some detectable accumulation. Differences between littoral and pelagic zones has long been recognized, but what is generally believed is that macrophyte presence/carbon is always good to bacteria, a paradigm our data do not support.

The littoral zone of Lake Mangueira (especially in the north and south) is extensively colonized by emergent and submersed macrophytes, respectively, and these plants are expected to contribute large amounts of organic carbon to the system. However, our results showed that respiration in this zone can be lower than in the pelagic zone, at least during a part of the year. This may be the result of differences in DOC quality, mainly derived from macrophytes in the littoral zone and phytoplankton in the pelagic zone. The disappearance of these differences may be due to seasonality or to masking by mixing in this shallow, wind-dominated lake. The finding of lower respiration rates in the littoral zone means that DOC remains in the system, mostly in a low-molecular-weight, unreactive form. Hence, the general belief that macrophyte-derived carbon is always beneficial to bacteria is not supported. The littoral zone, therefore, shows a greater tendency to be a CO_2_ sink, compared to the pelagic zone. If lower respiration in littoral zones is a common feature of subtropical shallow lakes dominated by macrophytes, there may be important and still unrecognized implications for their global carbon metabolism. However important these implications may seem, important issues (e.g., organic carbon molecular size and quality spectra, effect of taxonomic structure of macrophytes, bacterial taxonomic and functional diversity, the role of sediment bacteria, and more experimental and field evidence) need to be addressed for a better understanding of the many ways the interaction between macrophytes and bacteria can impact global carbon metabolism of lakes.

## Conflict of Interest Statement

The authors declare that the research was conducted in the absence of any commercial or financial relationships that could be construed as a potential conflict of interest.
